# INTRA-ABDOMINAL DESMOID TUMOR WITH AN UNUSUAL ORIGIN IN THE INTESTINAL WALL: CASE REPORT

**DOI:** 10.1590/0102-672020180001e1410

**Published:** 2018-12-06

**Authors:** Tomas STICKAR, Juan Andrés Dárdano BERRIEL, Josep Lluis Molinero POLO, Yuhami Mitsahid Curbelo PEÑA, Julia Gardenyes MARTINEZ, Tonia Palau FIGUEROA, Jordi de Cozar DUCH, Manel Guixa GENER, Francesc Xavier Quer VALL, Helena Valverdu CARTIE

**Affiliations:** 1 Hospital Universitari de Vic, Vic, Barcelona , Spain.

**Keywords:** Desmoid tumor, Small bowel, Tumor desmoide, Intestino delgado

## INTRODUCTION

Desmoid tumors are a rare entity, histologically benign (fibroblastic proliferation) but with an infiltrative growth that gives them a local aggressive behavior^1^. The sporadic have an annual incidence of 2.4-4.6 per million inhabitants^1^, but their incidence increases in patients affected by familial adenomatous polyposis or Gardner’s syndrome. They are more frequent in women, and can be extra or intra-abdominal, the latter being the most frequent.

They affect the abdominal wall by 50%, retroperitoneum by 9% and the mesentery by 40%^2^. The description of tumors that depend on the intestinal wall is exceptional in the literature based on an exhaustive search in Pubmed and Cochrane with the key word “desmoid tumor small bowell” evidencing sporadic cases^2^.

## CASE REPORT

We present the case of a 76-year-old man with no pathological history. admitted in the emergency room due to a 5-day evolution fever associated with abdominal distension and a palpable mass in the hypogastrium. Hemodynamic instability with BP of 90/50 and tachycardia with good response to initial resuscitation with 2000 ml of physiological solution and antibiotic therapy (Metronidazole+Ceftriaxone). The exploration showed indurated and mobile formation in hypogastrium, without signs of peritoneal irritation. Blood analysis showed leukocytosis with immature cells, and CRP increased with normal lactate.

**FIGURE 1:**
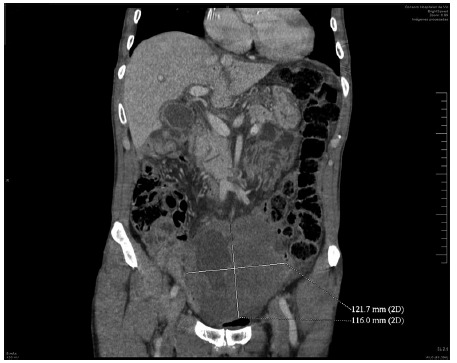
Abscess formation in the tumor and presence of hepatic intra-portal gas

Abdominal CT presented large supra-bladder pelvic mass of 12 cm with central necrosis and hydro-aerial level compatible with abscess formation in the tumor, presence of hepatic intra-portal gas in relation to the patient’s septic process (Figure1).

Pig tail drainage was placed obtaining purulent liquid, admission to the intensive care unit requiring noradrenaline (0.15 μg/kg/min). Improvement of the septic pattern occurred in the first 48 h with withdrawal of vasoactive drugs and decrease in inflammatory parameters. Cultures of blood and abscess liquid were positive for *Streptococcus anginosus* associated with mixed anaerobic flora. Percutaneous biopsy was negative for malignant cells; acute inflammatory component was associated with intestinal perforation.

At 72 h, orotracheal intubation was required due to progressive respiratory insufficiency, without any increase in inflammatory parameters. Thoracoabdominal CT demonstrated respiratory distress, abdominapelvic free fluid and completely drained intra-tumoral abscess.

Urgent surgical intervention showed a large tumor of 15x15 cm affecting the jejunum (20 cm from the duodenojejunal angle); intestinal resection was performed with free margins and lateral-lateral mechanical anastomosis was done. During the post-operative period, the patient recovered progressively and was discharged after 13 days.

Anatomopathological examination revealed mesenchymal proliferation on the intestinal wall without mucosa infiltration, constituted by a proliferation of elongated cells without pleomorphisms arranged forming bundles.

**FIGURE 2:**
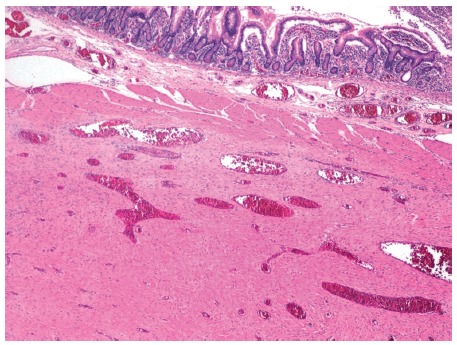
Mesenchymal proliferation in the intestinal wall below the muscle layer

Immunohistochemical analysis was negative for CD117, DOG1, ALK1, S100, CD34, desmin and actin and positive for vimentin and beta-catenin, desmoid tumor dependent on the jejunal wall, free neoplasic intestinal margins (Figure 2).

## DISCUSSION

Intra-abdominal desmoid tumors are frequently associated with familial adenomatous polyposis or Gardner’s syndrome. There are fewer than 100 published cases of sporadic intra-abdominal desmoid tumors^5^ such as ours but most depend on the mesentery; in this case the pathological anatomy, showed that the tumor was primary at intestinal wall. This presentation makes this case unusual.

The diagnosis of these tumors is made by immunohistochemistry.

The cells usually have a poorly circumscribed pattern with spindle cell proliferation forming long beams or spiral patterns; the cells do not show nuclear atypia or hyperchromasia are strongly positive to vimentin staining and the immunoreactivity to beta-catenin is expressed in the 67-80% of cases^1^ ^,^ ^7^.

Imaging exams are useful in establishing size, extension and anatomical relationship. One of the differential diagnoses to be taken into account are gastrointestinal stromal tumors (GIST) that share a stroma of common origin but are histologically, genetically and biologically different, so their treatment differs substantially^6^. Other differential diagnoses include solitary fibrous tumor, sclerosing mesenteritis, retroperitoneal fibrosis, retroperitoneal fibrosarcoma, carcinoid tumor and lymphoma^1^.

The therapeutic decision requires the approach of a multidisciplinary team. Surgery is considered the treatment of choice whenever possible^6^, other alternatives include radiotherapy, hormone therapy, treatment with NSAIDs and even observation.

Our case was presented as a complication that required urgent surgical treatment. However, whatever the treatment of choice, recurrence rates are high (30-40%)^8^. The follow-up must be done with physical examination and image test every 3-6 months during the first 2-3 years and then annually^9^.
